# Crystal structure of *catena*-poly[[[di­aqua­[1,2-bis(pyridin-4-yl)ethene]{4-[2-(pyridin-4-yl)ethen­yl]pyridinium}gold(I)iron(II)]-di-μ-cyanido] bis­[dicyanido­gold(I)] 1,2-bis­(pyridin-4-yl)ethene dihydrate]

**DOI:** 10.1107/S2056989020006738

**Published:** 2020-05-29

**Authors:** Sofiia V. Partsevska, Dina D. Naumova, Igor P. Matushko, Sergiu Shova, Il’ya A. Gural’skiy

**Affiliations:** a Department of Chemistry, Taras Shevchenko National University of Kyiv, Volodymyrska St. 64, Kyiv 01601, Ukraine; b Department of Inorganic Polymers, "Petru Poni" Institute of Macromolecular Chemistry, Romanian Academy of Science, Aleea Grigore Ghica Voda 41-A, Iasi 700487, Romania; c UkrOrgSyntez Ltd, Chervonotkatska St., 67, Kyiv 02094, Ukraine

**Keywords:** crystal structure, polymeric complex, iron(II) complex, di­cyano­aurate, aurophilic inter­actions

## Abstract

In the title compound [Fe(bpe)(Hbpe)Au(CN)_2_][Au(CN)_2_]_2_·bpe·2H_2_O}_*n*_ [where bpe = 1,2-bis­(pyridin-4-yl)ethene, C_12_H_10_N_2_], the Fe^II^ ion is coordinated in a distorted octa­hedral [FeN_4_O_2_] environment by two di­cyano­aurate anions, two water mol­ecules and two partially protonated 1,2-di(4-pyrid­yl)ethyl­ene mol­ecules. Di­cyano­aurate anions bridge the Fe^II^ cations, forming infinite chains, which propagate along the *a-*axis direction. The chains are connected *via* aurophilic inter­actions with two non-coordinated di­cyano­aurate anions for each Fe^II^ ion. The polymeric chains inter­act with each other *via* π–π stacking between the guest bpe mol­ecules and multiple hydrogen bonds.

## Abstract 2

In the title compound, the Fe^II^ ion is coordinated in a distorted octa­hedral [FeN_4_O_2_] environment by two di­cyano­aurate anions, two water mol­ecules and two partially protonated 1,2-di(4-pyrid­yl)ethyl­ene mol­ecules. Di­cyano­aurate anions bridge the Fe^II^ cations, forming infinite chains, which propagate along the *a-*axis direction. The chains are connected *via* aurophillic inter­actions with two non-coordinated di­cyano­aurate anions for each Fe^II^ ion.

## Chemical context   

Iron(II) complexes exhibiting spin-crossover (SCO) properties attract considerable attention because of their fascinating ability to change multiple physical properties (magnetic, optical, mechanical, *etc*.) under the influence of external stimuli (Gütlich & Goodwin, 2004 ▸). These materials can be integrated into various devices as switches, triggers, chemical sensors, *etc.* (Suleimanov *et al.*, 2015 ▸). For these reasons, new SCO materials, which undergo transition with defined temperature, hysteresis and abruptness are strongly desired. There are several classical approaches as how to modulate the SCO characteristics of complexes, among them the introduction of slightly modified ligands and co-ligands to obtain new SCO compounds and inclusion of some guest mol­ecules to already existing complexes (Ni *et al.*, 2017 ▸).

Fe^II^ Hofmann clathrate (Hofmann & Höchtlen, 1903 ▸) analogues represent one of the biggest classes of SCO coord­ination compounds. They are cyano­bimetallic complexes of general formula [Fe(*L*)_*n*_{*M*(CN)_*x*_}_*y*_] in which the Fe^II^ ions are connected by bridging cyano­metallic anions into infinite layers (*n* = 2 for monodentate ligands and *n* = 1 for bis-monodentate ligands). These layers are supported by N-donor aromatic ligands (*L* = pyridine, diazines and their substituted analogues). Di-, tetra- and octa­cyano­metallic (*x* = 2, *y* = 2: *M* = Cu, Ag, Au; *x* = 4, *y* = 1: *M* = Ni, Pd, Pt) anions have been introduced to develop coordination compounds of this kind. It has been shown that the inclusion of guest mol­ecules can significantly influence the temperature, completeness and step character of spin transition in complexes belonging to this class (Ohtani & Hayami, 2017 ▸). In order to develop new SCO Hofmann clathrate analogues with voids big enough to incorporate bulky guest mol­ecules, some bis-monodentate pyridine-based ligands have been introduced, such as 4,4′-bi­pyridine (Yoshida *et al.*, 2013 ▸), bis­(4-pyrid­yl)acetyl­ene (Bartual-Murgui *et al.*, 2011 ▸), bis­(4-pyrid­yl)ethyl­ene (Muñoz-Lara *et al.*, 2012 ▸), *etc*.
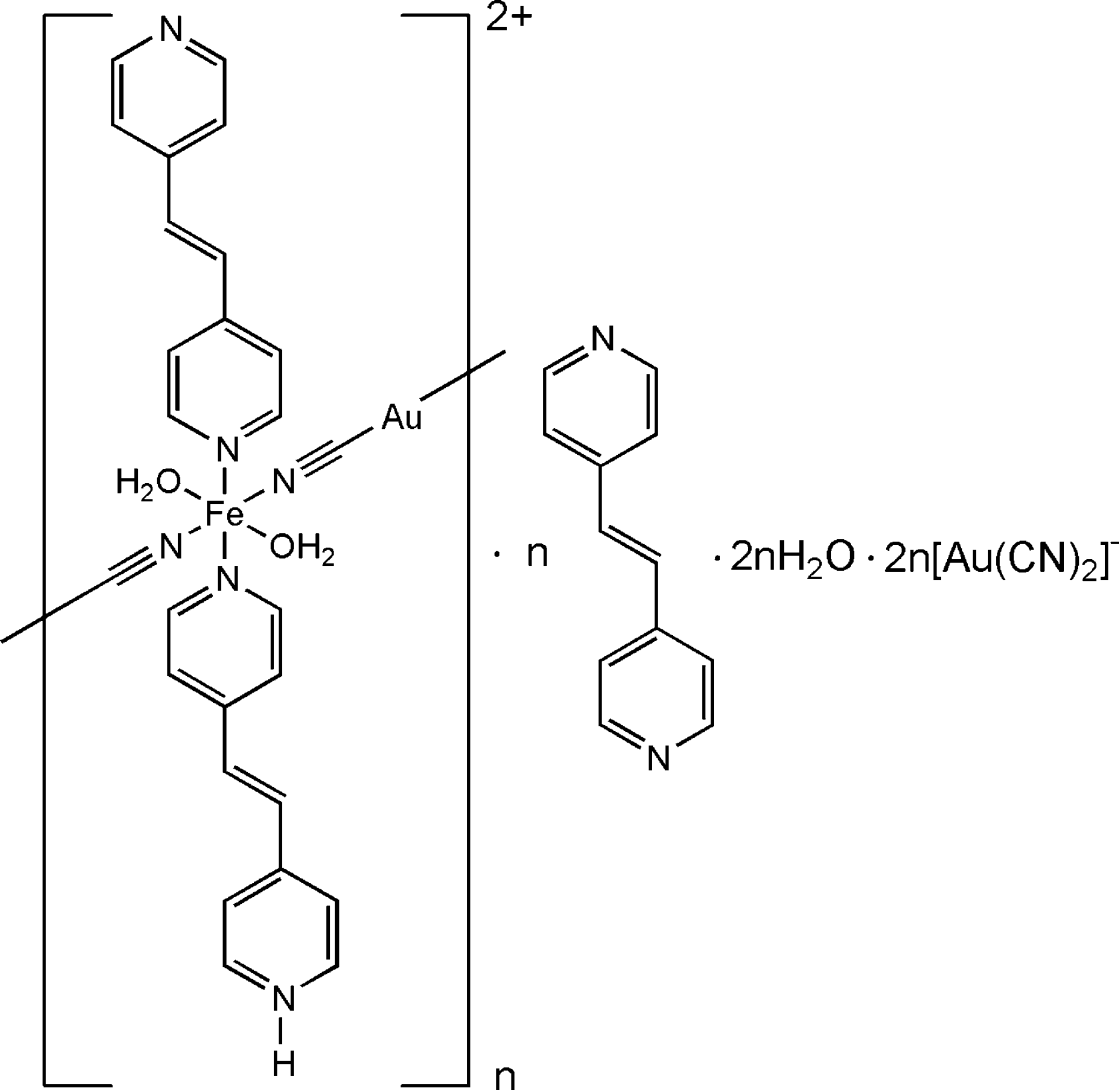


Here we describe the crystal structure of a new cyano­metallic Fe^II^ complex with bpe of general formula [Fe(bpe)(Hbpe)Au(CN)_2_](Au(CN)_2_)_2_·bpe·2H_2_O in which the Fe^II^ ions are stabilized in the high-spin (HS) state.

## Structural commentary   

**Figure 1:**
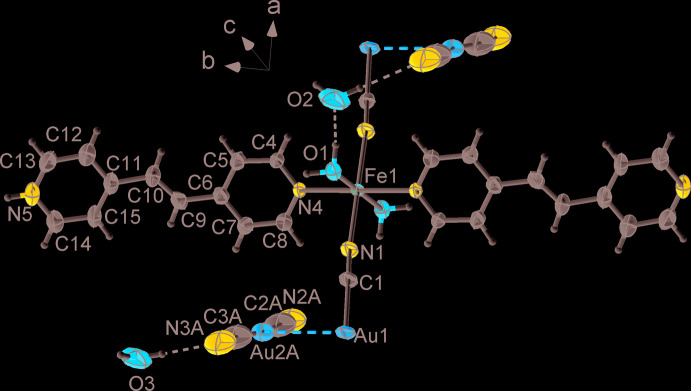
A fragment of the crystal structure of the title compound showing the atom-labelling scheme. Displacement ellipsoids are drawn at the 50% probability level. The guest bpe mol­ecule and disorder of the [Au(CN)_2_]^−^ counter-ions are not shown for clarity. Symmetry-generated atoms are not labelled.

The title compound crystallizes in the triclinic *P* 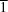 space group. The iron(II) ion has a distorted [FeN_4_O_2_] octa­hedral environment (Fig. 1 ▸) formed by two mol­ecules of 1,2-bis­(4-pyrid­yl)ethyl­ene (bpe) [Fe1—N4 = 2.223 (6) Å], two mol­ecules of water [Fe1–O1 = 2.081 (5) Å] and two cyano bridges [Fe1—N1 = 2.180 (6) Å]. Notably, the N5 atoms of the coordinated bpe mol­ecules are protonated (with 0.5 occupancy of each H atom). These N5 atoms create hydrogen bonds with symmetry-generated N5 atoms of bpe mol­ecules from the neighbouring chain [N5⋯N5^i^ = 2.677 (14) Å, N5—H5*A*⋯N5^i^ = 176°; Table 1 ▸].

The deviation from the ideal octa­hedral geometry of the Fe^II^ coordination environment is Σ|90 −  θ| = 6.8°, where θ are *cis*-N—Fe—N or *cis*-N—Fe—O angles. Two CN^−^ anions bridge the Fe^2+^ and Au^+^ cations [Fe1⋯Au1 = 5.280 (3) Å], creating a one-dimensional polymer, Fe1—N1—C1 = 172.8 (7)°, N1—C1—Au1 = 179.1 (8)° and C1—Au1—C1 = 180.0°, leading to a very slight deviation from linearity of the chains. This chain binds one guest bpe and two guest water mol­ecules per Fe^II^ centre.

## Supra­molecular features   

**Figure 2:**
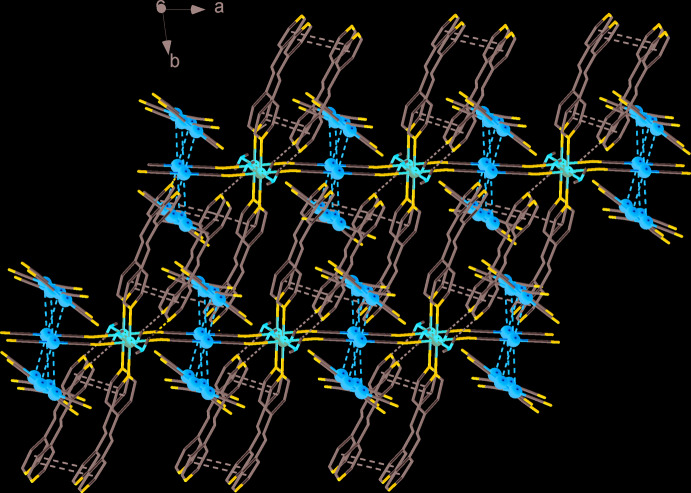
A view along the *c* axis of the crystal packing of the title compound. π–π contacts and hydrogen bonds are shown as black dashed lines. Aurophilic inter­actions are shown as orange dashed lines. Guest water mol­ecules are omitted for clarity.

**Figure 3:**
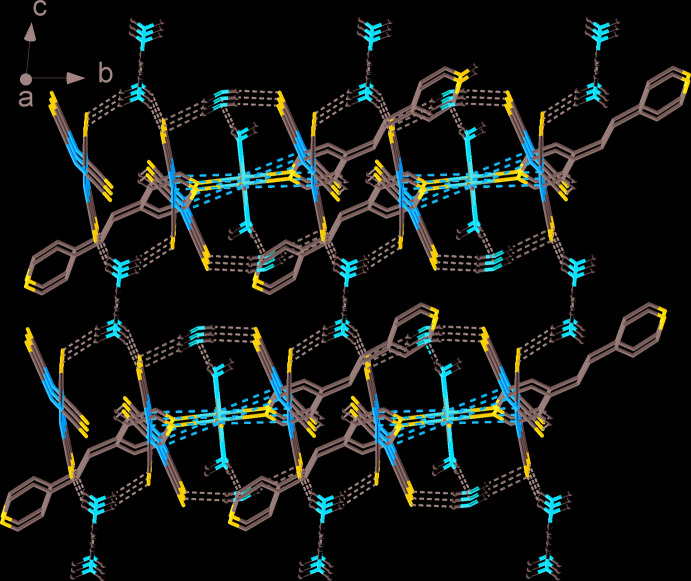
A view along the *a* axis of the crystal packing showing the network of hydrogen bonds as black dashed lines. H atoms not involved in hydrogen bonding and guest bpe mol­ecules are omitted for clarity.

The structure is characterized by the presence of several different kinds of weak inter­actions that create a three-dimensional supra­molecular framework. Two free [Au(CN)_2_]^−^ counter-ions are connected with the polymeric chains by aurophilic inter­actions and C7⋯N2*B* hydrogen bonds [C7⋯N2*B* = 2.78 (3) Å, C7—H7⋯N2*B* = 132°]. These free counter-ions are disordered over two positions with Au1—Au2*A* = 3.324 (1) Å and Au1—Au2*B* = 3.101 (5) Å. The polymeric chains are connected to each other *via* π–π inter­actions (Fig. 2 ▸) between the coordinated and guest mol­ecules of bpe (*Cg*1⋯*Cg*2 = 3.650 (5) Å, α = 10.3°, offset = 1.043 Å, where *Cg*1 and *Cg*2 are the centroids of the N4/C4–C8 and N6/C16–C20 rings, respectively, and *Cg*3⋯*Cg*4 = 3.794 (6) Å, α = 6.8°, offset = 1.835 Å, where *Cg*3 and *Cg*4 are the centroids of N5/C11–C15 and N6^ii^/ C^ii^16–C20 rings, respectively]. Guest bpe mol­ecules are additionally linked to the polymeric chains by hydrogen bonds with coordinated water mol­ecules [O1⋯N6 = 2.736 (9) Å, O1—H1*B*⋯N6 = 158°]. One of the guest water mol­ecules forms hydrogen bonds with the coord­inated water [Fig. 3 ▸; O2⋯O1 = 2.744 (13) Å, O1—H1*A*⋯O2 = 156°] and weak hydrogen bonds with free di­cyano­aurate counter-ions [O2⋯N2*A* ^ii^ = 3.45 (7) Å, O2—H2*A*⋯N2*A* ^ii^ = 179°; O2⋯N3*B* ^iii^ = 3.37 (3) Å, O2—H2*B*⋯N3*B* ^iii^ = 172°. The O3 guest water atom is bound to another symmetry-generated counterpart [O3⋯O3^v^ = 2.69 (4) Å, O3—H3*C*⋯O3^v^ = 179°] and free di­cyano­aurate counter-ions [O3⋯N3*A* = 2.99 (3) Å, O3—H3*B*⋯N3*A* = 167°; O3⋯N2*A* ^iv^ = 2.98 (4) Å, O3—H3*A*⋯N2*A* ^iv^ = 159°]. Hydrogen-bonding parameters are summarized in Table 1 ▸.

## Database survey   

A search of the Cambridge Structural Database (CSD version 5.40, last update February 2019; Groom *et al.*, 2016 ▸) revealed that the current structure has never been published before. 101 cyano­metallic structures containing Fe—N≡C—Au fragments were found. These hits include multiple temperature-dependant measurements, which were conducted to study the spin-crossover characteristics of Fe^II^ complexes. For example, these hits include a three-dimensional framework *catena*-[tetra­(μ-cyano)(μ-pyrazine)­irondigold] (IRIKUR01–IRIKUR09; Gural’skiy *et al.*, 2016 ▸). One particular compound resembles the title MOF: *catena*-[bis­(μ-cyano)­bis­(2-phenyl­pyrazine)­bis­(aqua)­iron(II)gold(I) bis­(cyano)­gold(I)] (MOJ­FEZ; Kucheriv *et al.*, 2019 ▸).

## Synthesis and crystallization   

Crystals of the title compound were prepared by the slow diffusion method between three layers in a 3 ml tube. The first layer was a solution of K[Au(CN)_2_] (0.03 mmol) in water (0.5 ml), the second was a mixture of water/ethanol (1:2, 1.5 ml) and the third layer was a solution of 1,2-di(4-pyrid­yl)ethyl­ene (0.05 mmol) and [Fe(OTs)_2_]·6H_2_O (0.01 mmol; OTs = *p*-toluene­sulfonate) in ethanol (0.5 ml) with 0.2 ml of water. After two weeks, red crystals grew in the second layer; the crystals were collected and kept in the mother solution prior to measurement.

## Refinement   

Crystal data, data collection and structure refinement details are summarized in Table 2 ▸. The hydrogen atoms were placed at their expected calculated positions (C—H = 0.93, N—H = 0.86, O—H = 0.92–0.96 Å) and refined as riding for the guest water mol­ecules (O2, O3) and aromatic rings, and as rotating for the coordinated water mol­ecule (O1) with *U_i_*_so_(H) = 1.2*U*_iso_(C), *U*_iso_(H) = 1.2*U*_iso_(N), *U*_iso_(H) = 1.5*U*_iso_(O). *U*_aniso_ values for all C and N atoms in the guest di­cyano­aurate anions and the O2 and O3 water mol­ecules were constrained to be equal using the EADP command. Distances N3*A*—C3*A* and N2*A*—C2*A* were restrained to a target of 1.15 Å and distances Au2*A*—C3*A* and Au2*A*—C2*A* were restrained to a target of 1.99 Å using the DFIX command. The following distances were restrained to be equal using the SADI command: C2*A*—N2*A* and C2*B*—N2*B*; Au1—C2*A* and Au1—C2*B*; C3*A*—N3*A* and C3*B*—N3*B*; Au1—C3*A* and Au1—C3*B*; C2*A*—C3*A* and C2*B*—C3*B*.

## Supplementary Material

Crystal structure: contains datablock(s) I. DOI: 10.1107/S2056989020006738/tx2022sup1.cif

Structure factors: contains datablock(s) I. DOI: 10.1107/S2056989020006738/tx2022Isup2.hkl

CCDC reference: 2004716

Additional supporting information:  crystallographic information; 3D view; checkCIF report

## Figures and Tables

**Table 1:** Hydrogen-bond geometry (Å, °)

*D*—H⋯*A*	*D*—H	H⋯*A*	*D*⋯*A*	*D*—H⋯*A*
O1—H1*A*⋯O2	0.93	1.86	2.744 (13)	156
O1—H1*B*⋯N6	0.93	1.85	2.736 (9)	158
C7—H7⋯N2*B*	0.93	2.08	2.78 (3)	132
N5—H5*A*⋯N5^i^	0.86	1.82	2.677 (14)	176
O2—H2*A*⋯N2*A* ^ii^	0.96	2.49	3.45 (7)	179
O2—H2*B*⋯N3*B* ^iii^	0.94	2.43	3.37 (3)	172
O3—H3*B*⋯N3*A*	0.92	2.08	2.99 (3)	167
O3—H3*A*⋯N2*A* ^iv^	0.92	2.11	2.98 (4)	159
O3—H3*C*⋯O3^v^	0.94	1.75	2.69 (4)	179

Symmetry codes: (i) 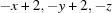; (ii) 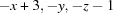; (iii) 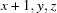; (iv) 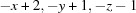; (v) 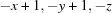.

**Table 2:** Experimental details

Crystal data	
Chemical formula	[AuFe(C_12_H_11_N_2_)(CN)_2_(C_12_H_10_N_2_)][Au(CN)_2_]_2_·C_12_H_10_N_2_·2H_2_O
*M*_r_	1422.60
Crystal system, space group	Triclinic, *P* 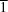
Temperature (K)	293
*a*, *b*, *c* (Å)	10.5601 (7), 11.0044 (12), 11.8145 (10)
α, β, γ (°)	80.212 (8), 69.124 (7), 78.565 (7)
*V* (Å^3^)	1249.9 (2)
*Z*	1
Radiation type	Mo *K*α
μ (mm^−1^)	9.11
Crystal size (mm)	0.4 × 0.3 × 0.2

Data collection	
Diffractometer	Rigaku Oxford Diffraction Xcalibur, Eos
Absorption correction	Multi-scan (*CrysAlis PRO*; Rigaku OD, 2018)
*T*_min_, *T*_max_	0.350, 1.000
No. of measured, independent and observed [*I* > 2σ(*I*)] reflections	9662, 4402, 3540
*R*_int_	0.042
(sin θ/λ)_max_ (Å^−1^)	0.595

Refinement	
*R*[*F* ^2^ > 2σ(*F* ^2^)], *wR*(*F* ^2^), *S*	0.050, 0.156, 1.04
No. of reflections	4402
No. of parameters	272
No. of restraints	9
H-atom treatment	H-atom parameters constrained
Δρ_max_, Δρ_min_ (e Å^−3^)	2.27, −1.04

Computer programs: *CrysAlis PRO* (Rigaku OD, 2018 ▸), *SHELXT2014/5* (Sheldrick, 2015*a* ▸), *SHELXL* (Sheldrick, 2015*b* ▸) and *OLEX2* (Dolomanov *et al.*, 2009 ▸).

## supplementary crystallographic information

### Crystal data

**Table:**

[AuFe(C_12_H_11_N_2_)(CN)_2_(C_12_H_10_N_2_)][Au(CN)_2_]_2_·C_12_H_10_N_2_·2H_2_O	*Z* = 1
*M**_r_* = 1422.60	*F*(000) = 670
Triclinic, *P*1	*D*_x_ = 1.890 Mg m^−^^3^
*a* = 10.5601 (7) Å	Mo *K*α radiation, λ = 0.71073 Å
*b* = 11.0044 (12) Å	Cell parameters from 3572 reflections
*c* = 11.8145 (10) Å	θ = 1.9–27.9°
α = 80.212 (8)°	µ = 9.11 mm^−^^1^
β = 69.124 (7)°	*T* = 293 K
γ = 78.565 (7)°	Block, red
*V* = 1249.9 (2) Å^3^	0.4 × 0.3 × 0.2 mm

### Data collection

**Table:**

Rigaku Oxford Diffraction Xcalibur, Eos diffractometer	3540 reflections with *I* > 2σ(*I*)
Detector resolution: 8.0797 pixels mm^-1^	*R*_int_ = 0.042
ω scans	θ_max_ = 25.0°, θ_min_ = 1.9°
Absorption correction: multi-scan (CrysAlisPro; Rigaku OD, 2018)	*h* = −11→12
*T*_min_ = 0.350, *T*_max_ = 1.000	*k* = −13→12
9662 measured reflections	*l* = −11→14
4402 independent reflections	

### Refinement

**Table:**

Refinement on *F*^2^	9 restraints
Least-squares matrix: full	Hydrogen site location: mixed
*R*[*F*^2^ > 2σ(*F*^2^)] = 0.050	H-atom parameters constrained
*wR*(*F*^2^) = 0.156	*w* = 1/[σ^2^(*F*_o_^2^) + (0.0855*P*)^2^ + 0.8591*P*] where *P* = (*F*_o_^2^ + 2*F*_c_^2^)/3
*S* = 1.03	(Δ/σ)_max_ < 0.001
4402 reflections	Δρ_max_ = 2.27 e Å^−^^3^
272 parameters	Δρ_min_ = −1.04 e Å^−^^3^

### Special details

**Table:**

Geometry. All esds (except the esd in the dihedral angle between two l.s. planes) are estimated using the full covariance matrix. The cell esds are taken into account individually in the estimation of esds in distances, angles and torsion angles; correlations between esds in cell parameters are only used when they are defined by crystal symmetry. An approximate (isotropic) treatment of cell esds is used for estimating esds involving l.s. planes.

### Fractional atomic coordinates and isotropic or equivalent isotropic displacement parameters (Å^2^)

**Table:**

	*x*	*y*	*z*	*U*_iso_*/*U*_eq_	Occ. (<1)
Au1	1.000000	0.000000	−0.500000	0.0441 (2)	
Fe1	1.500000	0.000000	−0.500000	0.0292 (4)	
N6	1.2335 (8)	0.1426 (7)	−0.1913 (6)	0.0501 (18)	
O1	1.4274 (5)	−0.0394 (6)	−0.3113 (5)	0.0438 (14)	
H1A	1.496163	−0.065149	−0.276444	0.066*	
H1B	1.381652	0.030289	−0.270914	0.066*	
C8	1.3795 (8)	0.2828 (8)	−0.5220 (7)	0.0423 (19)	
H8	1.353283	0.259226	−0.581414	0.051*	
C5	1.4616 (8)	0.3566 (8)	−0.3576 (8)	0.043 (2)	
H5	1.495858	0.380110	−0.303466	0.052*	
C4	1.5067 (7)	0.2397 (8)	−0.3988 (7)	0.0395 (19)	
H4	1.570087	0.185167	−0.369306	0.047*	
C18	1.0834 (8)	0.3621 (9)	−0.0954 (8)	0.048 (2)	
N4	1.4646 (6)	0.2017 (6)	−0.4772 (5)	0.0317 (14)	
C21	0.9996 (10)	0.4782 (10)	−0.0484 (8)	0.055 (2)	
H21	0.942320	0.523726	−0.089865	0.066*	
C9	1.2973 (8)	0.5573 (8)	−0.3485 (8)	0.044 (2)	
H9	1.240584	0.609573	−0.386971	0.053*	
C17	1.1829 (10)	0.2903 (10)	−0.0506 (8)	0.059 (3)	
H17	1.201690	0.315099	0.012449	0.071*	
N1	1.2923 (6)	0.0142 (7)	−0.5030 (6)	0.0433 (17)	
C19	1.0669 (9)	0.3182 (10)	−0.1910 (8)	0.052 (2)	
H19	1.003778	0.363330	−0.226531	0.063*	
C20	1.1418 (9)	0.2094 (9)	−0.2342 (9)	0.055 (2)	
H20	1.126215	0.181889	−0.297643	0.065*	
C6	1.3638 (8)	0.4388 (8)	−0.3989 (7)	0.0412 (19)	
C11	1.2282 (9)	0.7084 (8)	−0.1962 (8)	0.048 (2)	
C7	1.3274 (8)	0.4002 (8)	−0.4855 (7)	0.0415 (19)	
H7	1.267286	0.453324	−0.519895	0.050*	
C15	1.1322 (10)	0.7868 (9)	−0.2320 (9)	0.057 (3)	
H15	1.116876	0.775000	−0.301887	0.069*	
C10	1.3093 (9)	0.5968 (8)	−0.2560 (8)	0.047 (2)	
H10	1.375191	0.550547	−0.224182	0.057*	
N5	1.0744 (8)	0.9079 (7)	−0.0674 (7)	0.0513 (19)	
H5A	1.025191	0.968647	−0.026905	0.062*	0.5
C1	1.1866 (8)	0.0083 (8)	−0.5027 (7)	0.0413 (19)	
C13	1.1698 (10)	0.8351 (10)	−0.0344 (9)	0.061 (3)	
H13	1.185868	0.851036	0.033766	0.073*	
C12	1.2496 (10)	0.7344 (9)	−0.0949 (9)	0.057 (2)	
H12	1.317168	0.684662	−0.067302	0.069*	
C16	1.2541 (9)	0.1809 (10)	−0.1014 (9)	0.058 (3)	
H16	1.319389	0.132921	−0.069841	0.070*	
C14	1.0551 (11)	0.8852 (10)	−0.1667 (9)	0.064 (3)	
H14	0.987822	0.937040	−0.193066	0.077*	
N3A	0.7442 (6)	0.2821 (9)	−0.2440 (3)	0.140 (5)	0.8
C3A	0.8293 (4)	0.2875 (6)	−0.3312 (2)	0.140 (5)	0.8
Au2A	0.96785 (10)	0.30552 (9)	−0.49275 (9)	0.1010 (3)	0.8
C2A	1.1004 (4)	0.3499 (6)	−0.6389 (3)	0.140 (5)	0.8
N2A	1.1797 (6)	0.3786 (9)	−0.7294 (4)	0.140 (5)	0.8
N3B	0.703 (3)	0.119 (3)	−0.1529 (17)	0.140 (5)	0.2
C3B	0.7686 (18)	0.1679 (19)	−0.2399 (12)	0.140 (5)	0.2
Au2B	0.8855 (5)	0.2557 (4)	−0.3973 (6)	0.151 (2)	0.2
C2B	1.010 (2)	0.3636 (18)	−0.4940 (14)	0.140 (5)	0.2
N2B	1.095 (3)	0.424 (3)	−0.552 (2)	0.140 (5)	0.2
O2	1.5706 (15)	−0.1345 (17)	−0.1543 (12)	0.109 (5)	0.6
H2A	1.640962	−0.201711	−0.187906	0.164*	0.6
H2B	1.609492	−0.067311	−0.147186	0.164*	0.6
O3	0.574 (2)	0.503 (3)	−0.1194 (19)	0.109 (5)	0.4
H3B	0.617417	0.427938	−0.148163	0.164*	0.2
H3A	0.637317	0.556568	−0.154373	0.164*	0.4
H3C	0.522617	0.501478	−0.035993	0.164*	0.2

### Atomic displacement parameters (Å^2^)

**Table:**

	*U*^11^	*U*^22^	*U*^33^	*U*^12^	*U*^13^	*U*^23^
Au1	0.0232 (2)	0.0500 (3)	0.0655 (4)	−0.00604 (19)	−0.0212 (2)	−0.0086 (2)
Fe1	0.0194 (7)	0.0277 (9)	0.0434 (8)	−0.0019 (6)	−0.0110 (6)	−0.0127 (6)
N6	0.056 (4)	0.038 (5)	0.048 (4)	−0.004 (4)	−0.005 (4)	−0.012 (3)
O1	0.041 (3)	0.045 (4)	0.044 (3)	0.005 (3)	−0.013 (3)	−0.014 (3)
C8	0.041 (4)	0.041 (5)	0.047 (5)	−0.003 (4)	−0.014 (4)	−0.015 (4)
C5	0.042 (4)	0.036 (5)	0.054 (5)	−0.009 (4)	−0.013 (4)	−0.017 (4)
C4	0.034 (4)	0.035 (5)	0.055 (5)	0.000 (3)	−0.019 (4)	−0.020 (4)
C18	0.037 (4)	0.045 (6)	0.051 (5)	−0.013 (4)	0.003 (4)	−0.011 (4)
N4	0.029 (3)	0.024 (4)	0.044 (4)	0.000 (3)	−0.014 (3)	−0.011 (3)
C21	0.052 (5)	0.048 (6)	0.065 (6)	−0.003 (4)	−0.014 (5)	−0.024 (5)
C9	0.035 (4)	0.031 (5)	0.065 (6)	0.000 (3)	−0.016 (4)	−0.008 (4)
C17	0.064 (6)	0.072 (8)	0.046 (5)	−0.012 (5)	−0.016 (5)	−0.021 (5)
N1	0.030 (3)	0.037 (4)	0.066 (5)	−0.004 (3)	−0.018 (3)	−0.011 (3)
C19	0.052 (5)	0.059 (7)	0.042 (5)	−0.006 (5)	−0.011 (4)	−0.009 (4)
C20	0.054 (5)	0.051 (6)	0.057 (6)	−0.010 (5)	−0.010 (5)	−0.019 (5)
C6	0.031 (4)	0.041 (5)	0.050 (5)	−0.004 (3)	−0.008 (3)	−0.014 (4)
C11	0.048 (5)	0.037 (5)	0.055 (5)	−0.009 (4)	−0.006 (4)	−0.019 (4)
C7	0.042 (4)	0.036 (5)	0.051 (5)	−0.005 (4)	−0.021 (4)	−0.008 (4)
C15	0.071 (6)	0.041 (6)	0.065 (6)	0.009 (5)	−0.027 (5)	−0.029 (5)
C10	0.056 (5)	0.034 (5)	0.055 (5)	−0.003 (4)	−0.018 (4)	−0.016 (4)
N5	0.056 (5)	0.039 (5)	0.053 (4)	−0.004 (4)	−0.006 (4)	−0.020 (4)
C1	0.026 (4)	0.043 (5)	0.054 (5)	−0.005 (3)	−0.012 (4)	−0.008 (4)
C13	0.058 (6)	0.064 (7)	0.066 (6)	−0.014 (5)	−0.016 (5)	−0.031 (5)
C12	0.064 (6)	0.047 (6)	0.065 (6)	−0.004 (5)	−0.022 (5)	−0.021 (5)
C16	0.047 (5)	0.057 (7)	0.062 (6)	0.008 (5)	−0.014 (5)	−0.014 (5)
C14	0.075 (7)	0.046 (7)	0.076 (7)	0.010 (5)	−0.033 (6)	−0.024 (5)
N3A	0.181 (11)	0.132 (9)	0.155 (11)	0.018 (9)	−0.130 (10)	−0.026 (9)
C3A	0.181 (11)	0.132 (9)	0.155 (11)	0.018 (9)	−0.130 (10)	−0.026 (9)
Au2A	0.1098 (6)	0.0872 (7)	0.1463 (8)	0.0284 (5)	−0.0985 (6)	−0.0475 (5)
C2A	0.181 (11)	0.132 (9)	0.155 (11)	0.018 (9)	−0.130 (10)	−0.026 (9)
N2A	0.181 (11)	0.132 (9)	0.155 (11)	0.018 (9)	−0.130 (10)	−0.026 (9)
N3B	0.181 (11)	0.132 (9)	0.155 (11)	0.018 (9)	−0.130 (10)	−0.026 (9)
C3B	0.181 (11)	0.132 (9)	0.155 (11)	0.018 (9)	−0.130 (10)	−0.026 (9)
Au2B	0.174 (4)	0.092 (3)	0.276 (6)	0.037 (3)	−0.186 (5)	−0.081 (4)
C2B	0.181 (11)	0.132 (9)	0.155 (11)	0.018 (9)	−0.130 (10)	−0.026 (9)
N2B	0.181 (11)	0.132 (9)	0.155 (11)	0.018 (9)	−0.130 (10)	−0.026 (9)
O2	0.101 (10)	0.146 (14)	0.087 (8)	0.033 (9)	−0.066 (7)	−0.010 (8)
O3	0.101 (10)	0.146 (14)	0.087 (8)	0.033 (9)	−0.066 (7)	−0.010 (8)

### Geometric parameters (Å, º)

**Table:**

Au1—C1^i^	1.979 (7)	N1—C1	1.130 (10)
Au1—C1	1.979 (7)	C19—H19	0.9300
Au1—Au2A	3.3243	C19—C20	1.366 (13)
Au1—Au2A^i^	3.3243	C20—H20	0.9300
Au1—Au2B	3.101 (4)	C6—C7	1.367 (11)
Au1—Au2B^i^	3.101 (4)	C11—C15	1.336 (13)
Fe1—O1^ii^	2.081 (5)	C11—C10	1.479 (11)
Fe1—O1	2.081 (5)	C11—C12	1.379 (12)
Fe1—N4	2.223 (6)	C7—H7	0.9300
Fe1—N4^ii^	2.223 (6)	C15—H15	0.9300
Fe1—N1^ii^	2.180 (6)	C15—C14	1.385 (12)
Fe1—N1	2.181 (6)	C10—H10	0.9300
N6—C20	1.294 (12)	N5—H5A	0.8600
N6—C16	1.307 (12)	N5—C13	1.290 (12)
O1—H1A	0.9319	N5—C14	1.330 (12)
O1—H1B	0.9285	C13—H13	0.9300
C8—H8	0.9300	C13—C12	1.385 (13)
C8—N4	1.320 (10)	C12—H12	0.9300
C8—C7	1.377 (11)	C16—H16	0.9300
C5—H5	0.9300	C14—H14	0.9300
C5—C4	1.387 (11)	N3A—C3A	1.1014
C5—C6	1.401 (12)	C3A—Au2A	1.9543
C4—H4	0.9300	Au2A—C2A	1.8554
C4—N4	1.315 (9)	C2A—N2A	1.1381
C18—C21	1.472 (13)	N3B—C3B	1.1270
C18—C17	1.390 (13)	C3B—Au2B	2.0356
C18—C19	1.377 (12)	Au2B—C2B	1.8767
C21—C21^iii^	1.317 (17)	C2B—N2B	1.1712
C21—H21	0.9300	O2—H2A	0.9640
C9—H9	0.9300	O2—H2B	0.9427
C9—C6	1.466 (11)	O3—H3B	0.9225
C9—C10	1.298 (11)	O3—H3A	0.9170
C17—H17	0.9300	O3—H3C	0.9389
C17—C16	1.393 (13)		
			
C1^i^—Au1—C1	180.0	C16—C17—H17	120.4
C1^i^—Au1—Au2A	98.7 (3)	C1—N1—Fe1	172.8 (7)
C1—Au1—Au2A	81.3 (3)	C18—C19—H19	119.5
C1—Au1—Au2A^i^	98.7 (14)	C20—C19—C18	121.0 (9)
C1^i^—Au1—Au2A^i^	81.3 (14)	C20—C19—H19	119.5
C1^i^—Au1—Au2B	88.0 (3)	N6—C20—C19	123.2 (9)
C1—Au1—Au2B	92.0 (3)	N6—C20—H20	118.4
C1^i^—Au1—Au2B^i^	92 (3)	C19—C20—H20	118.4
C1—Au1—Au2B^i^	88 (3)	C5—C6—C9	122.6 (7)
Au2A^i^—Au1—Au2A	180.0 (18)	C7—C6—C5	116.4 (8)
Au2B—Au1—Au2B^i^	180 (5)	C7—C6—C9	120.9 (8)
O1^ii^—Fe1—O1	180.0	C15—C11—C10	124.7 (8)
O1^ii^—Fe1—N4^ii^	89.1 (2)	C15—C11—C12	116.4 (9)
O1—Fe1—N4	89.1 (2)	C12—C11—C10	118.9 (9)
O1^ii^—Fe1—N4	90.9 (2)	C8—C7—H7	119.9
O1—Fe1—N4^ii^	90.9 (2)	C6—C7—C8	120.1 (8)
O1—Fe1—N1^ii^	90.4 (2)	C6—C7—H7	119.9
O1—Fe1—N1	89.6 (2)	C11—C15—H15	119.5
O1^ii^—Fe1—N1^ii^	89.6 (2)	C11—C15—C14	121.1 (9)
O1^ii^—Fe1—N1	90.4 (2)	C14—C15—H15	119.5
N4—Fe1—N4^ii^	180.0	C9—C10—C11	126.2 (9)
N1^ii^—Fe1—N4	89.6 (2)	C9—C10—H10	116.9
N1^ii^—Fe1—N4^ii^	90.4 (2)	C11—C10—H10	116.9
N1—Fe1—N4^ii^	89.6 (2)	C13—N5—H5A	121.4
N1—Fe1—N4	90.4 (2)	C13—N5—C14	117.3 (8)
N1^ii^—Fe1—N1	180.0	C14—N5—H5A	121.4
C20—N6—C16	118.2 (8)	N1—C1—Au1	179.0 (8)
Fe1—O1—H1A	114.1	N5—C13—H13	118.2
Fe1—O1—H1B	113.4	N5—C13—C12	123.5 (9)
H1A—O1—H1B	100.1	C12—C13—H13	118.2
N4—C8—H8	118.2	C11—C12—C13	119.6 (9)
N4—C8—C7	123.6 (7)	C11—C12—H12	120.2
C7—C8—H8	118.2	C13—C12—H12	120.2
C4—C5—H5	120.5	N6—C16—C17	123.0 (9)
C4—C5—C6	119.0 (7)	N6—C16—H16	118.5
C6—C5—H5	120.5	C17—C16—H16	118.5
C5—C4—H4	118.2	C15—C14—H14	119.0
N4—C4—C5	123.5 (8)	N5—C14—C15	122.1 (9)
N4—C4—H4	118.2	N5—C14—H14	119.0
C17—C18—C21	124.5 (8)	N3A—C3A—Au2A	174.7
C19—C18—C21	120.2 (9)	C3A—Au2A—Au1	88.63 (19)
C19—C18—C17	115.4 (9)	C2A—Au2A—Au1	101.1 (2)
C8—N4—Fe1	121.6 (5)	C2A—Au2A—C3A	169.8
C4—N4—Fe1	120.0 (5)	N2A—C2A—Au2A	178.6
C4—N4—C8	117.1 (7)	N3B—C3B—Au2B	179.57 (10)
C18—C21—H21	117.8	C3B—Au2B—Au1	89.4 (6)
C21^iii^—C21—C18	124.4 (12)	C2B—Au2B—Au1	105.7 (7)
C21^iii^—C21—H21	117.8	C2B—Au2B—C3B	156.3
C6—C9—H9	116.6	N2B—C2B—Au2B	174.6
C10—C9—H9	116.6	H2A—O2—H2B	110.8
C10—C9—C6	126.9 (8)	H3B—O3—H3A	104.8
C18—C17—H17	120.4	H3A—O3—H3C	120.1
C18—C17—C16	119.2 (9)		

Symmetry codes: (i) −*x*+2, −*y*, −*z*−1; (ii) −*x*+3, −*y*, −*z*−1; (iii) −*x*+2, −*y*+1, −*z*.

### Hydrogen-bond geometry (Å, º)

**Table:**

*D*—H···*A*	*D*—H	H···*A*	*D*···*A*	*D*—H···*A*
O1—H1*A*···O2	0.93	1.86	2.744 (13)	156
O1—H1*B*···N6	0.93	1.85	2.736 (9)	158
C7—H7···N2*B*	0.93	2.08	2.78 (3)	132
N5—H5*A*···N5^iv^	0.86	1.82	2.677 (14)	176
O2—H2*A*···N2*A*^ii^	0.96	2.49	3.45 (7)	179
O2—H2*B*···N3*B*^v^	0.94	2.43	3.37 (3)	172
O3—H3*B*···N3*A*	0.92	2.08	2.99 (3)	167
O3—H3*A*···N2*A*^vi^	0.92	2.11	2.98 (4)	159
O3—H3*C*···O3^vii^	0.94	1.75	2.69 (4)	179

Symmetry codes: (ii) −*x*+3, −*y*, −*z*−1; (iv) −*x*+2, −*y*+2, −*z*; (v) *x*+1, *y*, *z*; (vi) −*x*+2, −*y*+1, −*z*−1; (vii) −*x*+1, −*y*+1, −*z*.

## References

[bb1] Bartual-Murgui, C., Ortega-Villar, N. A., Shepherd, H. J., Muñoz, M. C. C., Salmon, L., Molnár, G., Bousseksou, A. & Real, J. A. (2011). *J. Mater. Chem.* **21**, 7217–7222.

[bb2] Dolomanov, O. V., Bourhis, L. J., Gildea, R. J., Howard, J. A. K. & Puschmann, H. (2009). *J. Appl. Cryst.* **42**, 339–341.

[bb3] Groom, C. R., Bruno, I. J., Lightfoot, M. P. & Ward, S. C. (2016). *Acta Cryst.* B**72**, 171–179. 10.1107/S2052520616003954 PMC4822653 27048719

[bb4] Gural’skiy, I. A., Golub, B. O., Shylin, S. I., Ksenofontov, V., Shepherd, H. J., Raithby, P. R., Tremel, W. & Fritsky, I. O. (2016). *Eur. J. Inorg. Chem.* pp. 3191–3195.

[bb5] Gütlich, P. & Goodwin, H. A. (2004). *Spin Crossover in Transition Metal Compounds I*, pp. 1–47. Berlin, Heidelberg: Springer-Verlag.

[bb6] Hofmann, K. A. & Höchtlen, F. (1903). *Ber. Dtsch. Chem. Ges.* **36**, 1149–1151.

[bb7] Kucheriv, O. I., Barakhtii, D. D., Malinkin, S. O., Shova, S. & Gural’skiy, I. A. (2019). *Acta Cryst.* E**75**, 1149–1152. 10.1107/S2056989019009678 PMC6690477 31417782

[bb8] Muñoz-Lara, F. J., Gaspar, A. B., Muñoz, M. C., Arai, M., Kitagawa, S., Ohba, M. & Real, J. A. (2012). *Chem. Eur. J.* **18**, 8013–8018. 10.1002/chem.201200377 22628190

[bb9] Ni, Z.-P., Liu, J.-L., Hoque, M. N., Liu, W., Li, J.-Y., Chen, Y.-C. & Tong, M.-L. (2017). *Coord. Chem. Rev.* **335**, 28–43.

[bb10] Ohtani, R. & Hayami, S. (2017). *Chem. Eur. J.* **23**, 2236–2248. 10.1002/chem.201601880 27417666

[bb16] Rigaku OD (2016). *CrysAlis PRO*. Rigaku Oxford Diffraction, Yarnton, England.

[bb11] Sheldrick, G. M. (2015*a*). *Acta Cryst* A**71**, 3–8.

[bb12] Sheldrick, G. M. (2015*b*). *Acta Cryst.* C**71**, 3–8.

[bb13] Suleimanov, I., Kraieva, O., Sánchez Costa, J., Fritsky, I. O., Molnár, G., Salmon, L. & Bousseksou, A. (2015). *J. Mater. Chem. C.* **3**, 5026–5032.

[bb14] Yoshida, K., Akahoshi, D., Kawasaki, T., Saito, T. & Kitazawa, T. (2013). *Polyhedron*, **66**, 252–256.

